# Exome Sequencing Reveals Novel and Recurrent Mutations with Clinical Significance in Inherited Retinal Dystrophies

**DOI:** 10.1371/journal.pone.0116176

**Published:** 2014-12-29

**Authors:** María González-del Pozo, Cristina Méndez-Vidal, Nereida Bravo-Gil, Alicia Vela-Boza, Joaquin Dopazo, Salud Borrego, Guillermo Antiñolo

**Affiliations:** 1 Department of Genetics, Reproduction and Fetal Medicine, Institute of Biomedicine of Seville, University Hospital Virgen del Rocío/CSIC/University of Seville, Seville, Spain; 2 Centro de Investigación Biomédica en Red de Enfermedades Raras (CIBERER), Seville, Spain; 3 Genomics and Bioinformatics Platform of Andalusia (GBPA), Seville, Spain; 4 Department of Bioinformatics, Prince Felipe Research Centre (CIPF), Valencia, Spain; 5 Functional Genomics Node (INB), Prince Felipe Research Centre (CIPF), Valencia, Spain; National Eye Institute, United States of America

## Abstract

This study aimed to identify the underlying molecular genetic cause in four Spanish families clinically diagnosed of Retinitis Pigmentosa (RP), comprising one autosomal dominant RP (adRP), two autosomal recessive RP (arRP) and one with two possible modes of inheritance: arRP or X-Linked RP (XLRP). We performed whole exome sequencing (WES) using NimbleGen SeqCap EZ Exome V3 sample preparation kit and SOLID 5500xl platform. All variants passing filter criteria were validated by Sanger sequencing to confirm familial segregation and the absence in local control population. This strategy allowed the detection of: (i) one novel heterozygous splice-site deletion in *RHO*, c.937-2_944del, (ii) one rare homozygous mutation in *C2orf71*, c.1795T>C; p.Cys599Arg, not previously associated with the disease, (iii) two heterozygous null mutations in *ABCA4,* c.2041C>T; p.R681* and c.6088C>T; p.R2030*, and (iv) one mutation, c.2405-2406delAG; p.Glu802Glyfs*31 in the ORF15 of *RPGR*. The molecular findings for *RHO* and *C2orf71* confirmed the initial diagnosis of adRP and arRP, respectively, while patients with the two *ABCA4* mutations, both previously associated with Stargardt disease, presented symptoms of RP with early macular involvement. Finally, the X-Linked inheritance was confirmed for the family with the *RPGR* mutation. This latter finding allowed the inclusion of carrier sisters in our preimplantational genetic diagnosis program.

## Introduction

Inherited Retinal Dystrophies (IRDs) are a group of pathologies characterized by progressive dysfunction and death of retinal photoreceptors. Retinitis Pigmentosa (RP [MIM 268000]) is the most common form of IRD, affecting 1∶3,000 to 4,000 individuals worldwide [1], [2]. Typical RP is characterized by early loss of rod photoreceptors followed by secondary loss of cone photoreceptors. The clinical features of RP include night blindness, peripheral constriction of the visual field and pigment spicule deposits in the midperiphery of the retina. The condition may segregate as an autosomal dominant (adRP), autosomal recessive (arRP), or an X-linked trait (XLRP) [3]. The autosomal recessive form of RP is the most common worldwide, accounting for approximately 39% of cases in Spain [4]. RP is genetically heterogeneous, with mutations in more than 60 genes and loci identified to date (RetNet - Retinal Information Network, http://www.sph.uth.tm.edu/Retnet/). The contribution of most of the genes to the overall prevalence of RP is relatively small, and for the majority, only one or a few families carrying the same pathogenic mutations have been reported worldwide [5].

The methods and tools available for molecular diagnosis have evolved greatly over the last years. Genotyping microarrays of known mutations for each phenotype have been used effectively to diagnose a percentage of families [6], but this approach is unable to detect novel mutations and results particularly ineffective if clinical diagnosis is inaccurate or incomplete. Targeted sequencing of known genes has been a suitable diagnostic approach in diseases where the known causal genes explain almost all of the families. However, in diseases such as arRP, the genes identified to date hardly explain 50–70% of cases [7]. Therefore, a more efficient and higher throughput technique is required. Whole exome sequencing (WES) exhibit multiple advantages for new disease genes identification, allowing new phenotypic associations and rectifying apparent discrepancies between the molecular defect and clinical findings [8].

In this study, we conducted WES to uncover the genetic cause of RP in four Spanish families comprising one clinically diagnosed of adRP, two with a diagnosis of arRP and one with two possible modes of inheritance: arRP or XLRP. Our study revealed one novel heterozygous mutation in *RHO* (NM_000539.3), c.937-2_944del, in the adRP family, one rare homozygous mutation in *C2orf71* (NM_001029883.2), c.1795T>C; p.Cys599Arg, two known heterozygous mutations in *ABCA4* (NM_001034853.1), c.2041C>T; p.Arg681* and c.6088C>T; p.Arg2030*, and one known hemizygous mutation in *RPGR* (NM_001034853.1), c.2405-2406delAG; p.Glu802Glyfs*31. Family segregation analysis, frequency in control population and bioinformatic predictions supported the pathogenicity of the identified variants. These results further support WES as a powerful approach for genetic diagnosis of uncertain IRD cases.

## Materials and Methods

### Participants and clinical assessment

The study conformed to the tenets of the declaration of Helsinki (Edinburgh, 2000) and was approved by the Institutional Review Board of Hospital Virgen del Rocio, Seville. An informed consent form was signed by all participants for clinical and molecular genetic studies.

**Figure 1:**
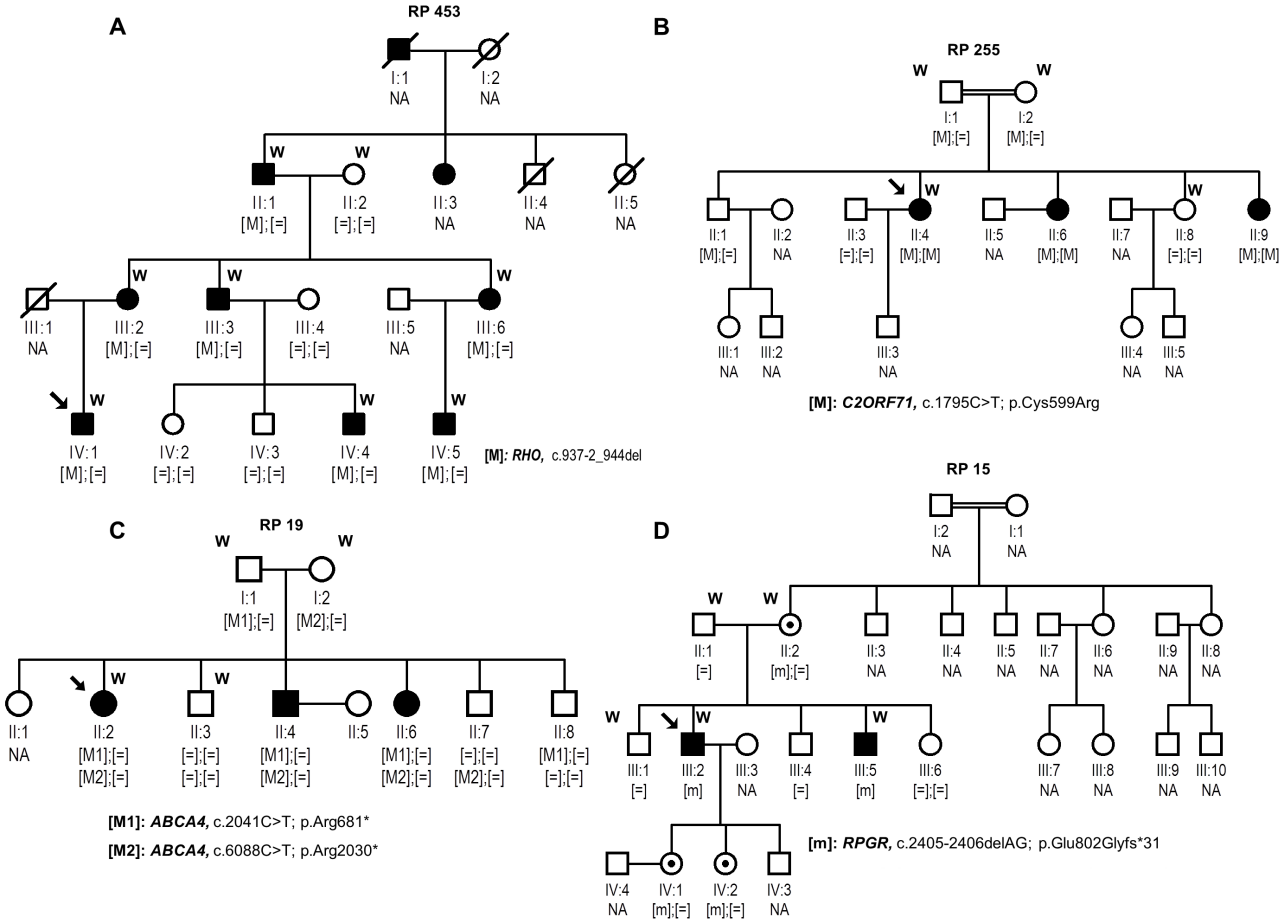
Segregation studies of identified variants in the four analyzed families. Below the individuals, genotypes are presented for each change segregating with RP. Index patients are indicated with a black arrow. [M];[M] represents homozygous mutants; [M];[ = ] indicates heterozygous carriers, [ = ];[ = ] indicates individuals carrying two wild-type alleles, whereas [m] represents hemizygous individuals. NA means non available DNA sample. W means samples processed by WES.

Our study involved four Spanish families comprising one clinically diagnosed of adRP (RP 453), two clinically diagnosed of arRP (RP 255, RP 19) and one with an ar/XLRP inheritance (RP15), all derived from the Ophthalmology Department to the Genetic, Reproduction and Fetal Medicine Department (Fig. 1). A panel of 200 healthy control individuals was also recruited.

Clinical diagnosis of RP was based on a full ophthalmic examination performed as described elsewhere [9]. All subjects underwent a peripheral blood extraction for genomic DNA isolation from leukocytes using standard protocols. A selection of DNA samples of each family were processed for WES (Fig. 1).

Prior to NGS, the index patients of the four families were first analyzed and excluded for a number of known mutations associated with adRP or arRP, by applying appropriate commercially available genotyping microarrays analysis (ASPER Ophthalmics, Tartu, Estonia). In addition, in the arRP families, the presence of mutations in *EYS* gene was excluded by direct Sanger sequencing of its 43 exons [10], and mutations in a selection of arRP genes were also excluded by a custom genome resequencing microarray experiment [9].

### Exome sequencing and data analysis

We performed exome sequencing using a modified Baylor College of Medicine protocol version 2.1 for sequencing on the SOLiD 5500xl platform as previously described [11], [12].

Filtering and prioritization of variants were carried out with the BiERapp tool (http://bierapp.babelomics.org) as follows. Variants which produce a synonymous change in the open reading frame and do not alter predicted splice sites were discarded. Then, variants present in NCBI dbSNP (www.ncbi.nlm.nih.gov/SNP/) and 1000 Genomes project databases (www.1000genomes.org/) with a MAF higher than 0.01 were eliminated. An additional filtering step was performed by discarding variants observed in a group of 267 healthy controls of the Spanish population (The Medical Genome Project, http://www.medicalgenomeproject.com/). Those genes presenting variants consistent with the inheritance pattern were considered as candidates. Finally, whole-exome data were further evaluated for second-site mutations acting as modifiers in other retinal disease genes (Retnet, https://sph.uth.edu/retnet/sum-dis.htm#A-genes).

### Verification and assessment of the pathogenicity of variants

Each predicted disease-causing variant was confirmed by Sanger sequencing and co-segregation analysis was performed in the rest of family members DNA samples. We used Polyphen-2 [13] (http://genetics.bwh.harvard.edu/pph2/), SIFT [14] (http://sift.jcvi.org/) and MutationTaster [15] (http://www.mutationtaster.org/) to evaluate the potential impact of novel missense substitutions on the function of the encoded protein. We used the DiANNA web server for disulfide connectivity prediction [16] (http://clavius.bc.edu/~clotelab/DiANNA/). The correct name of the variation according to HGVS nomenclature guidelines was checked using Mutalyzer [17] (https://mutalyzer.nl/). Furthermore, splice site tool Prediction by Neural Network [18], [19], were applied to estimate the pathogenic nature of intronic sequence variants that could affect the splicing process. The HOPE server [20] (http://www.cmbi.ru.nl/hope/home) was used to analyze and predict the structural variations in mutant *C2orf71*.

In order to detect additional cases harboring novel mutations in *RHO* and *C2orf71,* we performed Sanger sequencing in those IRD families of our cohort without a molecular diagnosis. This study group was comprised of 282 families of which 92 were clinically diagnosed of arRP, 31 of adRP, 3 of cone rod dystrophies, 6 of Leber Congenital Amaurosis, 9 of Stargardt disease and 141 of sporadic RP.

## Results

### Clinical characteristics

Available clinical findings of the index patients and affected siblings of the four families are reported in Table 1. Molecular diagnosis of family RP 19 led to the clinical re-evaluation of the family since the identified *ABCA4* mutations were previously related to Stargardt disease. In this family, early symptoms including hemeralopy and decreased visual acuity indicated that the most suitable clinical diagnosis was RP with early macular involvement. In family RP 15, the scarce family information with two affected male siblings in one generation of the pedigree did not allow to adjust the mode of inheritance until the molecular diagnosis was achieved. The molecular findings for *RPGR* resulted in the clinical classification of this family as XLRP. The age of onset of symptoms (5–11 years), and the rest of clinical signs were similar to those previously reported in XLRP patients.

**Table 1:** Clinical characteristics of affected family members.

Family	Pedigree subject	*Gene* [Variant 1]; [Variant 2]	Onset age	First symptom	Age at time of the genetic assessment	Symptoms at time of the genetic assessment	Fundus examination	ERG scotopic/photopic
RP 453	IV:1 (index)	*RHO [c.937-2_944del];[ = ]*	10	Night blindness	33	Night blindness then visual field constriction leaving only the central 20° functional and preserved visual acuity. Photophobia.	Bone spicules in the periphery, optic disc pallor and preserved macula	N.A.
	III:2		7	Night blindness	57	Night blindness then visual field constriction leaving only 5° functional and decreased visual acuity (since age 35). Photophobia. Posterior subcapsular cataract.	Widespread deposition of bone-spicules, narrowed vessels.	N.A.
	III:3		6	Night blindness	54	Night blindness then visual field constriction and decreased visual acuity (since age 30). Epiretinal membrane (macular pucker) in one eye.	Widespread deposition of bone-spicules, optic disc pallor and narrowed vessels.	N.A.
	III:6		12	Night blindness	49	Night blindness then visual field constriction leaving only 15° functional and decreased visual acuity.	Diffuse hypopigmentation with bone spicules changes in the periphery. Preserved macula.	N.A.
	IV:4		9	Night blindness and decrease of visual field	13	Night blindness then visual field constriction leaving only 20° functional. Preserved visual acuity.	Bone spicule pigmentation in the periphery. Preserved macula.	N.A.
	IV:5		13	Night blindness	14	Night blindness	Some bone spicules in the periphery, preserved macula and optic disc. Narrowed vessels.	N.A.
RP 255#	II:4 (index)	*C2orf71* [p.Cys599Arg]; [p.Cys599Arg]	12	Night blindness	38	Night blindness, visual field constriction, decreased visual acuity, photophobia and abnormal color vision	Pale optic nerve disc, narrowed blood vessels and bone spicule pigmentation in the periphery	N.A.
RP 19	II:2 (index)	*ABCA4* [p.Arg681*]†; [p.Arg2030*]‡	4	Night blindness, decrease of visual acuity	32	Campimetry very altered and little assessable by the decrease of visual acuity, Night blindness	Waxy optic disc pallor, bone spicule pigmentation	N.R./N.R.
	II:4		5	Decrease of visual acuity	32	Campimetry not practicable dyschromatopsia	Waxy optic disc pallor, bone spicule pigmentation (areas not specified)	N.R./N.R.
	II:6		4	Decrease of visual acuity	30	Campimetry not practicable, dyschromatopsia	Waxy optic disc pallor, bone spicule pigmentation (areas not specified)	N.R./N.R.
RP 15	III:2 (index)	*RPGR* [p.Glu802Glyfs*31]¥ Hemizygous	5	Night blindness	29	Night blindness, visual field constriction, decreased visual acuity, nystagmus. Myopia	Bone spicule pigmentation in the periphery	N.A.
	III:5		11	Night blindness	23	Night blindness, visual field constriction, decreased visual acuity. Blue-yellow dyschromatopsia. Myopia	Bone spicules in the periphery, optic disc pallor and preserved macula	N.A.

# Consanguineous family

† Previously reported by Maugeri et al. [24] in a Stargardt patient in heterozygous state.

‡ Previously reported by Lewis et al. [25] in a Stargardt patient in heterozygous state.

¥ Previously reported by Vervoort et al. [23].

N.A.: Not available.

N.R.: Not recordable.

In the case of families RP 453 and RP 255 the initial diagnosis was confirmed. Affected members of family RP 453 had a history of night blindness from early childhood (ranging from 6–14 years old) and visual field loss before age 20. Later in the course of the disease, macular function was also severely compromised, leaving only residual central vision by the fourth decade of life. Index patient of family RP 255 reported night blindness as initial symptom at age 12, followed by progressive visual field constriction with relatively preserved central vision until late in the course of the disease. This patient manifested decreased visual acuity at age 30. Fundus examination showed typical signs of RP and ERG responses were not detectable for this patient, consistent with severe generalized rod cone dysfunction.

### Identification of mutations by whole exome sequencing

We performed WES of the selected members of each family using Roche NimbleGen SeqCap EZ Exome V3 sample preparation kit and SOLiD 5500xl. A mean of 40,341 SNVs per sample were found (S1 Table).

Filtering and data analysis with the BIERapp tool resulted in the identification of: (i) one novel heterozygous 10 bp deletion, c.937-2_944del, affecting the acceptor splice site of the exon 5 of *RHO* (ii) one rare homozygous mutation, c.1795T>C; p.Cys599Arg, in the exon 1 of *C2orf71*; (iii) two known heterozygous mutations, c.2041C>T; p.Arg681* and c.6088C>T; p.Arg2030*, in the exons 14 and 44 respectively, of *ABCA4* and (iv) one hemizygous mutation, c.2405-2406delAG; p.Glu802Glyfs*31 in exon ORF15 of *RPGR* (S1 Fig.), (Table 1). No other disease-causing variants or genetic modifiers were detected by WES in none of the analyzed families.

Interestingly, the mutation in *RPGR* is located in exon ORF15, a highly repetitive, hotspot mutation region, technically difficult to capture using standard exome enrichment protocols. Reads alignment of this region is shown in S1A Fig.

### Family segregation and assessment of the pathogenicity of the novel identified variants

Sanger sequencing of the available samples revealed that all variants identified by WES co-segregated with the disease phenotypes in the four families (Fig. 1). Mutations in *RPGR* and *ABCA4* genes have previously been associated with XLRP and Stargardt Disease, respectively. Additionally, novel variants in *RHO* and *C2orf71* were absent in 200 local control individuals. Importantly, although the variant in *C2orf71*, c.1795T>C; p.Cys599Arg was initially filtered as novel, when we screened the public Exome Variant Server database (EVS; http://evs.gs.washington.edu/EVS/), for the presence of this variant we found that it is not a novel, but rare mutation (dbSNP; rs377190272) observed in heterozygous state in 1 out of 6304 individuals, probably being an asymptomatic carrier.

**Figure 2:**
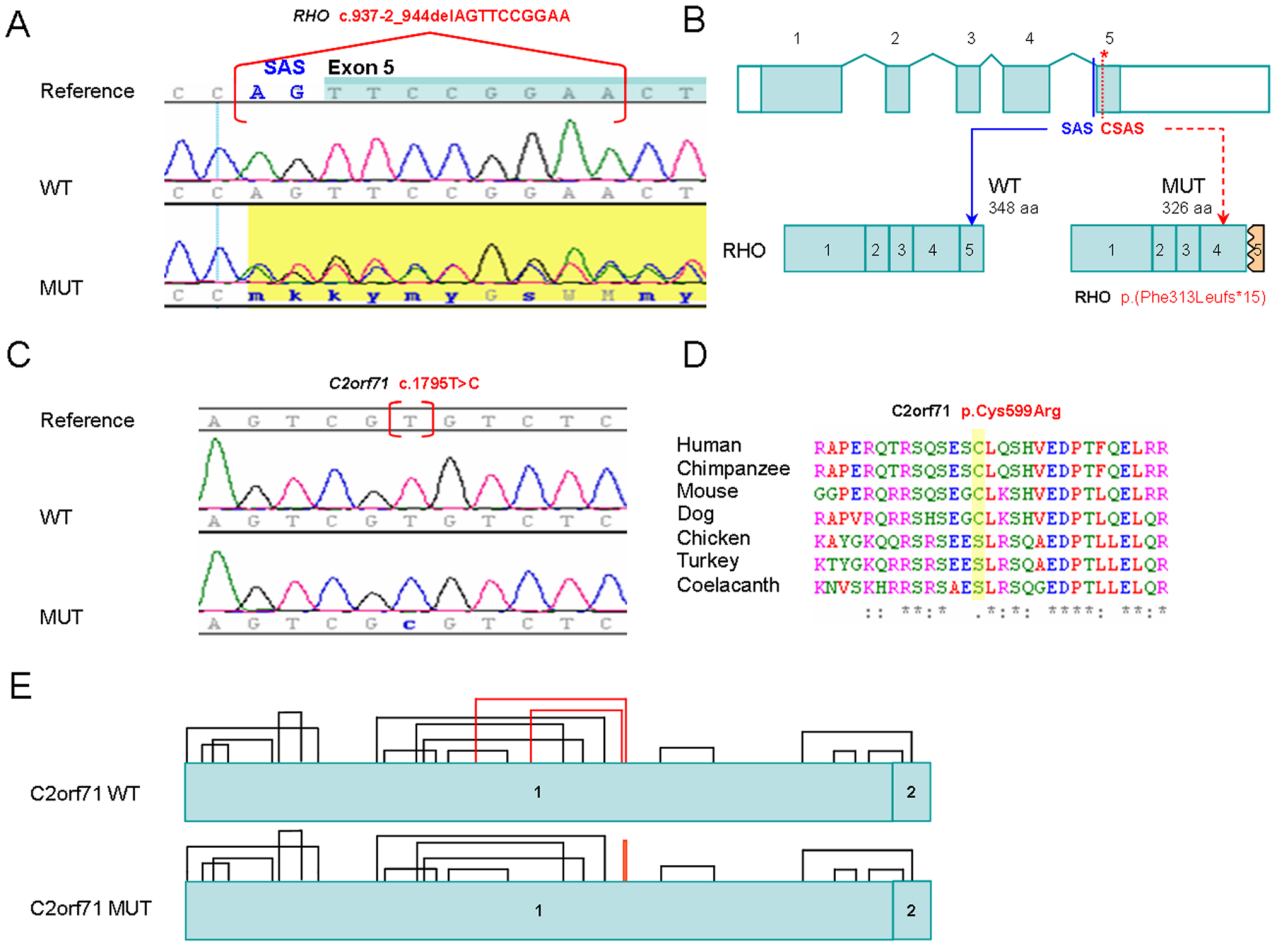
Novel pathogenic variants identified in this study. A) Chromatograms of wild type (WT) *RHO* DNA sequence (NM_000539.3) and RP subject (MUT) showing the heterozygous mutation c.937-2_944delAGTTCCGGAA. SAS: Splice Acceptor Site. Exon 5 is highlighted in blue. B) The *RHO* gene structure is composed of 5 exons that are indicated as filled boxes while 5′ and 3′ UTRs are shown as open boxes. The canonical SAS of exon 5 is in blue and the predicted cryptic SAS (CSAS) is in red and indicated with an asterisk. The WT protein product (left) has 348 aminoacids (aa) while the predicted mutant product (MUT) only 326 aa. C) Chromatograms of WT *C2orf71* (NM_001029883.2) DNA sequence and RP subject (MUT) showing the homozygous mutation c.1795T>C. D) Alignment of C2orf71 orthologous protein sequences showing conservation of the mutated residue p.Cys599Arg among mammals. E) Prediction of disulfide bonds formation for WT and mutant (MUT) C2orf71 proteins. According to the prediction, in the mutant protein structure, two of the disulfide bonds have been disrupted while a new one has been created. The disulfide bonds altered are in red.

In silico functional studies predicted that the deletion of 10 bp at position c.937-2 in *RHO* results in the loss of the canonical splice acceptor site (MutationTaster score: wild type allele/mutant allele = 0.94/-) in intron 4–5 and the use of a cryptic splice acceptor site downstream of the canonical splice acceptor site (MutationTaster score: 0.98) resulting in a truncated protein (Fig. 2A and 2B).

The variant in *C2orf71* (Fig. 2C), c.1795T>C; p.Cys599Arg relies on an evolutionary conserved region only in mammals (Fig. 2D). Moreover, in silico functional studies using DiANNA predicted that this variant affect the disulfide-bonding pattern. The cysteine at position 599 is expected to participate in the formation of a disulphide bond with other cysteine of the protein conditioning its proper folding (Fig. 2E). The HOPE tool [20] predicted that the original wild-type residue and the newly introduced mutant residue differ in size, charge, and hydrophobicity values.

Direct full sequencing of *RHO* and *C2orf71* genes in 282 additional patients without molecular diagnosis revealed no additional cases harboring c.937-2_944del or c.1795T>C in our cohort.

## Discussion

Next generation sequencing in families affected of Mendelian disorders has been a powerful tool for the identification of new disease genes and mutations. Other authors have recently used this approach to identify known and novel mutations in many RP families [21], [22]. Now, we show the achievement of the molecular diagnosis of four additional families clinically diagnosed of RP by exome sequencing. Diagnostic strategies used to date were focused in the screening of a limited number of genes for each phenotype and mode of inheritance. However, these approaches can fail to get the genetic characterization for the reasons outlined below.

Firstly, the information available in the medical history often does not allow the assignment of an accurate pattern of inheritance. Such is the case of family RP 15 which pedigree structure (Fig. 1) did not permit to discern between the two likely modes of inheritance, arRP and XLRP. As far as both daughters of the index patient (individuals IV:1 and IV:2) had reached the reproductive age, WES was performed to speed up a molecular diagnosis for these individuals. This resulted in the identification of a mutation (p.Glu802Glyfs*31) in *RPGR*, a gene associated with XLRP. This result allowed the two carrier sisters to be included in our preimplantational genetic diagnosis program. The mutation detected here, located in the exon ORF15 of *RPGR,* was previously identified by Vervoort et al. [23]. This highly repetitive region technically represents a challenge to capture protocols. However, the correct identification of the mutation showed the high effectiveness of the experimental system used.

Secondly, the clinical diagnosis is not often accurate since it is very difficult to assign a specific clinical diagnosis in both young and older subjects. Interestingly, in family RP 19, two null mutations in *ABCA4* were identified (p.Arg681* and p.Arg2030*). Although each of them were separately found in heterozygous state in subjects suffering from Stargardt disease [24], [25], in none of these cases the second mutation was identified. These results support the model of genotype-phenotype proposed by many researchers for mutations in *ABCA4* in which the combination of two null mutations results in a severe phenotype (like RP) [26]. The index patient, first came to our Department of Genetics at age 32 (Table 1) when the disease was at a very advanced stage. However, the onset of symptoms during the first decade of life, and the early macular involvement indicated that it is not a typical RP, considering most appropriate a diagnosis of RP with early macular involvement.

Given the high prevalence of carriers of deleterious mutations in RP genes among control population [27], and the growing number of experiments in which genetic variants are identified by NGS (EVS, 1000 genomes, 5000 genomes, etc), identifying real novel mutations is an increasingly difficult task. This approach allowed the identification of a rare, possibly pathogenic, missense variant in *C2orf71,* c.1795T>C (p.Cys599Arg), which was not found in 400 ethnically matched chromosomes. The fact that the variant in *C2orf71* was present in heterozygosity in only one control individual in EVS does not reduce the likelihood of being pathogenic, since the individual holding it could be an asymptomatic carrier. Other well known missense pathogenic variant such as p.M390R in *BBS1* is also present in the same database with an unexpected high frequency (26 heterozygous out of 6469 individuals, entry rs113624356). Moreover, this variant is predicted to affect the proper folding of the protein, partially conditioned by the disulfide-bonding pattern between cysteines. Although additional studies are needed to confirm the role of the rare variant identified in *C2orf71* in the pathogenesis of RP, segregation analysis and *in silico* prediction supports its pathogenicity. This type of clinically meaningful information of reported variants will facilitate the development of clinically relevant variants resources that are required for the complete transition of NGS to the clinic.

Furthermore, in the present study one novel heterozygous mutation has been identified in *RHO,* c.937-2_944del, segregating in a large adRP family with 9 affected members. The effect at the protein level is yet unclear. In silico tools predicted that the deletion of 10 bp at position c.937-2 in *RHO* results in the abolition of the canonical splice acceptor site in intron 4–5 and promotes the use of a cryptic splice site. The protein produced as a result would be missing the carboxyl terminal of rhodopsin p.(Phe313Leufs*15). Three different variants: c.937-1G>A (HGMD entry: CS941542) [28], c.937-1G>T (HGMD entry: CS961683) [29] and a 150 bp insertion replacing 30 bp of normal sequence (HGMD entry: CN942279) [30] also destroy the acceptor splice site of the intron 4 in three other adRP families. In studies of mRNA isolated from peripheral blood lymphocytes of an affected individual, the splice site mutation c.937-1G>A resulted in an aberrant rhodopsin mRNA processing and the missing of the last 36 amino acids of rhodopsin p.Phe313Lysfs*1 [31]. Furthermore, removal of 38 C-terminal amino acids (p.Lys311*) in a mutant form of bovine rhodopsin results in a misfolded protein which cannot bind 11-cisretinal or catalyse light-dependent activation of the rod cell G protein, transducin [32]. These studies may point to the mechanism by which the mutant rhodopsin in family RP 453 causes adRP. Clinically, affected members of this family manifested an early and severe retinal dysfunction with a rate of progression of disease higher than the above mentioned families with acceptor splice site mutations in intron 4 which produce dominant disease in a mild form [28], [29].

The results presented here have shown how in some cases it was difficult to detect the underlying genetic cause until the implementation of massive sequencing techniques. The problems associated with the clinical and genetic diagnosis of IRD affects directly to the patients who are likely to require a detailed explanation and genetic counselling. This work emphasizes the need to integrate the clinical phenotype, family history and genetic findings as a crucial step in the management of IRD patients and their relatives.

## Supporting Information

S1 Fig **Detection of** ***RPGR*** **ORF15 mutation.** A) View of reads alignment and coverage of c.2405-2406delAG variant in individual II:2 using IGV. The inserts refer to close-up views of the region harboring the mutated position (X: 38145845-47) in heterozygosis. B) Electropherograms of *RPGR* sequence from a control individual and from family members II:2 and III:2 showing the deletion in heterozygosis and homozygosis, respectively.
(TIF)

S1 Table **Quality of the exome data and variants identified in each individual.**
(XLS)
