# Correction: Exome Sequencing Reveals Novel and Recurrent Mutations with Clinical Significance in Inherited Retinal Dystrophies

**DOI:** 10.1371/journal.pone.0120166

**Published:** 2015-03-16

**Authors:** The PLOS ONE Staff

The first author’s name is incorrect in the citation. The correct short name is: González-del Pozo, M. The correct citation is: González-del Pozo, M, Méndez-Vidal C, Bravo-Gil N, Vela-Boza A, Dopazo J, Borrego S, et al. (2014) Exome Sequencing Reveals Novel and Recurrent Mutations with Clinical Significance in Inherited Retinal Dystrophies. PLoS ONE 9(12): e116176. doi:10.1371/journal.pone.0116176
