# Supplementary figures and images for: Exome Sequencing Reveals Novel and Recurrent Mutations with Clinical Significance in Inherited Retinal Dystrophies
Source: PLoS One. 2014 Dec 29;9(12):e116176. doi: 10.1371/journal.pone.0116176 (PMC4278866; doi:10.1371/journal.pone.0116176)

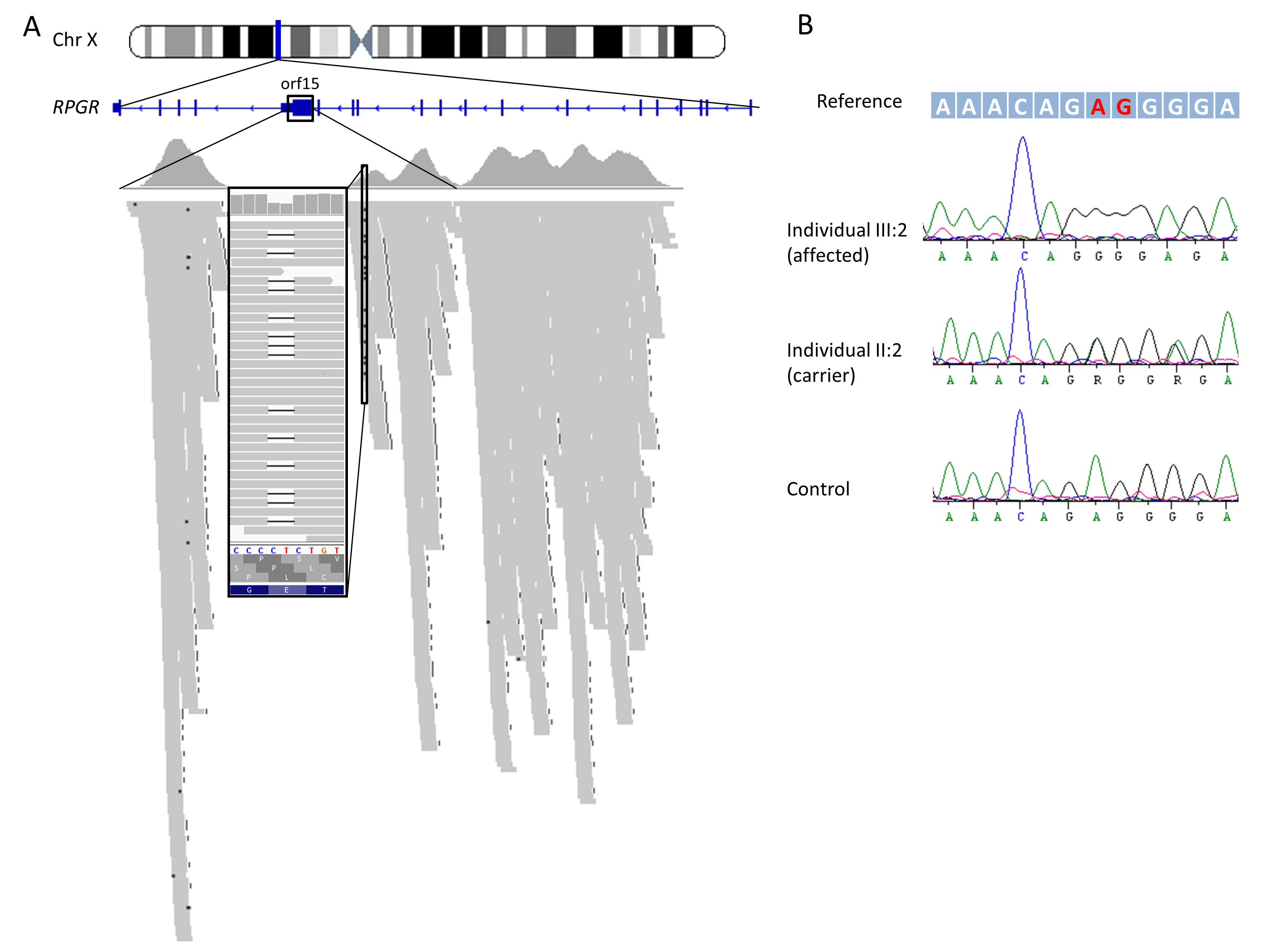

Supplement: S1 Fig — Detection of RPGR ORF15 mutation. A) View of reads alignment and coverage of c.2405-2406delAG variant in individual II:2 using IGV. The inserts refer to close-up views of the region harboring the mutated position (X: 38145845-47) in heterozygosis. B) Electropherograms of RPGR sequence from a control individual and from family members II:2 and III:2 showing the deletion in heterozygosis and homozygosis, respectively. (TIF) [file pone.0116176.s001.tif]
